# Safety and immunogenicity of a candidate tuberculosis vaccine MVA85A delivered by aerosol in BCG-vaccinated healthy adults: a phase 1, double-blind, randomised controlled trial

**DOI:** 10.1016/S1473-3099(14)70845-X

**Published:** 2014-08-20

**Authors:** Iman Satti, Joel Meyer, Stephanie A Harris, Zita-Rose Manjaly Thomas, Kristin Griffiths, Richard D Antrobus, Rosalind Rowland, Raquel Lopez Ramon, Mary Smith, Sharon Sheehan, Henry Bettinson, Helen McShane

**Affiliations:** a The Jenner Institute, University of Oxford, Oxford, UK; b Oxford Centre for Respiratory Medicine, The Churchill Hospital, Oxford, UK

## Abstract

**Background:**

Intradermal MVA85A, a candidate vaccine against tuberculosis, induces high amounts of Ag85A-specific CD4 T cells in adults who have already received the BCG vaccine, but aerosol delivery of this vaccine might offer immunological and logistical advantages. We did a phase 1 double-blind trial to compare the safety and immunogenicity of aerosol-administered and intradermally administered MVA85A

**Methods:**

In this phase 1, double-blind, proof-of-concept trial, 24 eligible BCG-vaccinated healthy UK adults were randomly allocated (1:1) by sequentially numbered, sealed, opaque envelopes into two groups: aerosol MVA85A and intradermal saline placebo or intradermal MVA85A and aerosol saline placebo. Participants, the bronchoscopist, and immunologists were masked to treatment assignment. The primary outcome was safety, assessed by the frequency and severity of vaccine-related local and systemic adverse events. The secondary outcome was immunogenicity assessed with laboratory markers of cell-mediated immunity in blood and bronchoalveolar lavage samples. Safety and immunogenicity were assessed for 24 weeks after vaccination. Immunogenicity to both insert Ag85A and vector modified vaccinia virus Ankara (MVA) was assessed by ex-vivo interferon-γ ELISpot and serum ELISAs. Since all participants were randomised and vaccinated according to protocol, our analyses were per protocol. This trial is registered with ClinicalTrials.gov, number NCT01497769.

**Findings:**

Both administration routes were well tolerated and immunogenic. Respiratory adverse events were rare and mild. Intradermal MVA85A was associated with expected mild local injection-site reactions. Systemic adverse events did not differ significantly between the two groups. Three participants in each group had no vaccine-related systemic adverse events; fatigue (11/24 [46%]) and headache (10/24 [42%]) were the most frequently reported symptoms. Ag85A-specific systemic responses were similar across groups. Ag85A-specific CD4 T cells were detected in bronchoalveolar lavage cells from both groups and responses were higher in the aerosol group than in the intradermal group. MVA-specific cellular responses were detected in both groups, whereas serum antibodies to MVA were only detectable after intradermal administration of the vaccine.

**Interpretation:**

Further clinical trials assessing the aerosol route of vaccine delivery are merited for tuberculosis and other respiratory pathogens.

**Funding:**

The Wellcome Trust and Oxford Radcliffe Hospitals Biomedical Research Centre.

## Introduction

Tuberculosis is caused by inhalation of *Mycobacterium tuberculosis* and remains a major global public health problem, causing 1·3 million deaths worldwide in 2012. ^1^ The only licensed vaccine, intradermal BCG, is effective in preventing disseminated tuberculosis, but confers a highly variable degree of protection against pulmonary tuberculosis, especially in adults.^2^, ^3^

One tuberculosis vaccine development strategy is to boost BCG with a subunit vaccine expressing one or more *M tuberculosis* antigens. An advanced candidate vaccine is modified vaccinia virus Ankara (MVA) expressing the highly conserved *M tuberculosis* antigen 85A (MVA85A). Given systemically, MVA85A induces potent Ag85A-specific CD4 T-cell responses in BCG-vaccinated adults.^4^

Inhalation of infectious droplets containing *M tuberculosis* bacilli is the most common natural route of *M tuberculosis* infection. Delivery of a tuberculosis vaccine directly to the respiratory mucosa might enhance local protective immune responses at the primary site of infection. Systemically administered BCG protects against systemic disease in childhood.^2^, ^5^ Airway vaccination might be needed to optimise protection against pulmonary disease. Studies in mice show that the location of antigen-specific cells in the airway is important for protection against *M tuberculosis* challenge.^6^ Recombinant MVA vectors delivered to mucosa induced high levels of mucosal immunity in animal models.^7^ Aerosol administration of MVA in non-human primates was safe and induced potent and long-lasting antigen-specific immunity.^8^ Aerosol delivery of a tuberculosis vaccine could offer logistic benefits over intradermal administration. This approach is feasible with aerosolised measles vaccines.^9^

In this proof-of-concept, double-blind, randomised phase 1 clinical trial, we compare the safety and immunogenicity of aerosolised and intradermally administered MVA85A in healthy BCG-vaccinated adults in the UK. This study is the first clinical evaluation of a candidate tuberculosis vaccine delivered by aerosol.

## Methods

### Study design and participants

Between Nov 10, 2011, and Nov 1, 2012, we enrolled 24 BCG-vaccinated, healthy adults 18–50 years of age. All participants had received their BCG vaccination at least 6 months before enrolment. Participants were recruited from the general population in Oxfordshire, UK, and seen and enrolled at the Centre for Clinical Vaccinology and Tropical Medicine in Oxford. Enrolled participants were in good health and had normal baseline haematology, coagulation, and biochemistry; a normal chest radiograph; no substantial abnormality of pulmonary function tests; and negative serological testing for hepatitis B, hepatitis C, and HIV. Latent *M tuberculosis* infection was excluded by a negative ex-vivo interferon-γ ELISpot response to *M tuberculosis* early secreted antigen of 6 kDa (ESAT-6) and 10-kDa culture filtrate protein (CFP-10) peptides. Current smokers, people using nasal or inhaled drugs, and those with a history of asthma or other respiratory disease were excluded. 38 people were initially screened and 14 were excluded (four withdrew consent and ten met exclusion criteria). All 24 enrolled participants were randomised, vaccinated, underwent bronchoscopy, and were followed up for 6 months after vaccination as per protocol.

All participants provided written informed consent. This phase 1 trial was approved by the Medicines and Healthcare Products Regulatory Agency (reference 2010-022381-27) and Oxfordshire Research Ethics Committee A (reference 11/SC/0021). This trial is registered with ClinicalTrials.gov, number NCT01497769.

### Randomisation and masking

Between Nov 10, 2011, and Nov 1, 2012, we randomly allocated eligible participants (1:1) to aerosol MVA85A (using the Omron MicroAir U22 ultrasonic mesh nebuliser [Omron Healthcare UK Ltd, Milton Keynes, UK]) and intradermal saline (group A) or intradermal MVA85A (delivered in a volume of 135 μL to the deltoid region of the arm by a 29G diameter, 12·7 mm length needle) and aerosol saline (group B). Randomisation was done with sequentially numbered, opaque, sealed envelopes opened by the study clinician at enrolment. Variable blocks (block sizes two to eight) in the randomisation sequence ensured allocation concealment. For safety reasons, the first participant was assigned to the new intervention (group A) with a 1 week delay before subsequent enrolments. Participants, the bronchoscopist, and immunologists were masked to treatment allocation, which was achieved through the paired-placebo trial design.

### Procedures

The vaccine used was clinical-grade MVA85A (lot number 010507), which was produced under good manufacturing practice conditions by IDT Biologika GmbH (Dessau-Rosslau, Germany).^4^ The planned dose of MVA85A was 1 × 10^8^ plaque-forming units (pfu), as used in previous trials. To help nebulisation, we used sterile 0·9% saline to make the 135 μL dose up to a volume of 1 mL. In the first two participants vaccinated by the aerosol route, this dose of MVA85A induced extremely powerful mucosal cellular immune responses. These responses at day 7 were higher than those previously recorded in non-human primates.^8^ In view of the potency of this route of administration, the protocol was amended by the principal investigator (and approved by the ethics committee and by the Medicines and Healthcare Products Regulatory Agency on Dec 22, 2011, and by the NHS Research and Development team on Jan 11, 2012) and the remaining 22 participants were vaccinated with MVA85A at a lower dose of 1 × 10^7^ pfu.

We did fibreoptic flexible bronchoscopies at 7 days after vaccination in accordance with British Thoracic Society Guidelines.^10^ After they had provided informed consent, participants fasted for 6 h without food and 2 h without water, and then received local anaesthesia and light sedation with fentanyl and midazolam. The bronchoscope was passed through the nose or mouth and local anaesthetic was delivered to the vocal cords and trachea. The bronchoscope was positioned in the bronchiole of the medial segment of the right middle lobe and 50 mL sterile 0·9% saline was injected and then aspirated into the lung segment. This bronchoalveolar lavage was repeated once and the samples placed in Falcon tubes (BD Biosciences [Oxford, UK]), which were transferred to Jenner Institute Laboratories (Oxford, UK) within 1 h. We measured vital signs, including peripheral oxygen saturation (S_p_O_2_), and monitored participants throughout the procedure. Participants were discharged home after 90 min.

We did the ex-vivo interferon-γ ELISpot assay on fresh peripheral blood mononuclear cells separated from whole blood as previously described.^11^ Samples were collected at baseline and at 1, 2, 4, 12, and 24 weeks after vaccination. Peripheral blood mononuclear cells (1 × 10^5^ per well and 3 × 10^5^ per well) were stimulated with one pool of 15-mer peptides spanning the length of Ag85A, MVA-CD4 and MVA-CD8 T-cell epitopes,^12^ a pool of ESAT-6, and a pool of CFP-10 peptides (Peptide Protein Research [Hampshire, UK]), and staphylococcal enterotoxin B from *Staphylococcus aureus* (Sigma-Aldrich [Gillingham, UK]).

For the intracellular cytokine staining, bronchoalveolar lavage cells were pelleted and stimulated at 1 × 10^6^ with 2 μg/mL Ag85A peptide pool; negative (unstimulated) and positive (staphylococcal enterotoxin B-stimulated) controls were included. We used 1 μg/mL of co-stimulatory αCD28 and αCD49d (BD Biosciences). Stimulated and unstimulated cells were incubated at 37°C in 5% CO_2_ for 2 h, after which Brefeldin-A (Sigma-Aldrich) was added before a further overnight incubation.

Whole blood was stimulated as previously described.^13^ Briefly, blood was incubated with 1 μg/mL αCD28, 1 μg/mL αCD49d (BD Biosciences) and stimulated with Ag85A peptide pool, staphylococcal enterotoxin B, or unstimulated. Samples were incubated at 37°C in 5% CO_2_ for 6 h, Brefeldin-A was added, and whole blood samples were incubated for another 6 h. Red blood cells were then lysed and samples were frozen for batched intracellular cytokine staining analysis.

We did surface and intracellular cytokine staining as previously described.^14^ Bronchoalveolar lavage cells were surface stained for viability (Molecular Probes LIVE/DEAD fixable stain, Invitrogen [Paisley, UK**]**), followed by CD4 (clone RPA-T4, Biolegend [London, UK]), CD14 (clone RMO52), and CD19 (clone J3-119) (Beckman Coulter [High Wycombe, UK]). Cells were stained intracellularly for CD3 (clone UCHT1), interferon-γ (clone 4S.B3, Ebiosciences [Hatfield, UK]), CD8 (clone SK1, BD Biosciences), interleukin-2 (clone N7.48A, Beckman Coulter), tumour necrosis factor (TNF)α (clone MAB11), and interleukin 17 (clone BL168, Biolegend).

Stimulated whole blood cells were permeabilised and stained as detailed previously. Samples were acquired on an LSR II flow cytometer (BD Biosciences). We analysed responses using FlowJo (Tree Star Inc [Ashland, USA]). We measured cytokines in live, singlet CD3, CD4, and CD8 T cells. The presented results are percentages of cytokine-positive cells minus the responses in unstimulated cells.

In ELISA, we assessed insert-specific and vector-specific humoral responses (IgG, IgA, and IgM) in serum samples collected on days 1, 2, 7, 14, 28, 84, and 168 and bronchoalveolar lavage fluid samples collected on day 7. Nunc Immunoplates were coated with recombinant Ag85A (Lionex [Braunschweig, Germany]) or wild-type MVA and incubated overnight. Plates were then blocked and diluted serum or neat bronchoalveolar lavage fluid samples were incubated. We used goat anti-human secondary antibody conjugated to alkaline phosphatase (Sigma) to detect antibodies, and plates were developed by the addition of substrate.

### Outcomes

The primary outcome of this trial was safety, which we assessed by the frequency and severity of vaccine-related local and systemic adverse events. Details of expected local skin adverse events (pain, erythema, and swelling), respiratory adverse events (cough, sore throat, wheeze, dyspnoea, sputum production, haemoptysis, chest pain, and appearance of bronchial mucosa), and systemic adverse events (fever, feverishness, fatigue, malaise, headache, myalgia, arthralgia, and nausea) were collected from participants by the use of a diary card, which the participants self-completed for 14 days after vaccination. We measured routine laboratory biochemical and haematological variables at 7 days and 84 days after vaccination. We issued participants with, and trained them in the use of, a handheld spirometer (Micro Spirometer, CareFusion [San Diego, CA, USA]) for home measurement of forced expiratory volume in 1 s (FEV_1_) and forced vital capacity (FVC), initially twice daily and then daily for 14 days after vaccination. The secondary outcome was immunogenicity to both insert (Ag85A) and vector (MVA) assessed with laboratory markers of cell-mediated immunity in blood and bronchoalveolar lavage samples by use of ex-vivo interferon-γ ELISpot, intracellular cytokine staining, and serum ELISAs for humoral responses.

### Statistical analysis

Safety data are summarised by the frequency and median number of adverse events per participant. We used the Mann-Whitney *U*-test to compare adverse events and immune responses between groups.

We did an area-under-the-curve (AUC) analysis to summate each participant's response over time. We used Mann-Whitney AUC to compare responses in both groups. Only results from the low-dose group were included in the analysis, because only two participants received the high dose and they both received vaccine by aerosol so it would be inappropriate to include those in the analysis. We judged p values less than 0·05 to be statistically significant. Prism Graph Pad Software version 5 was used for statistical analysis.

### Role of the funding source

The funder of the study had no role in study design, data collection, data analysis, data interpretation, or writing of the report. The corresponding author had full access to all the data in the study and had final responsibility for the decision to submit for publication.

## Results

**Figure 1:**
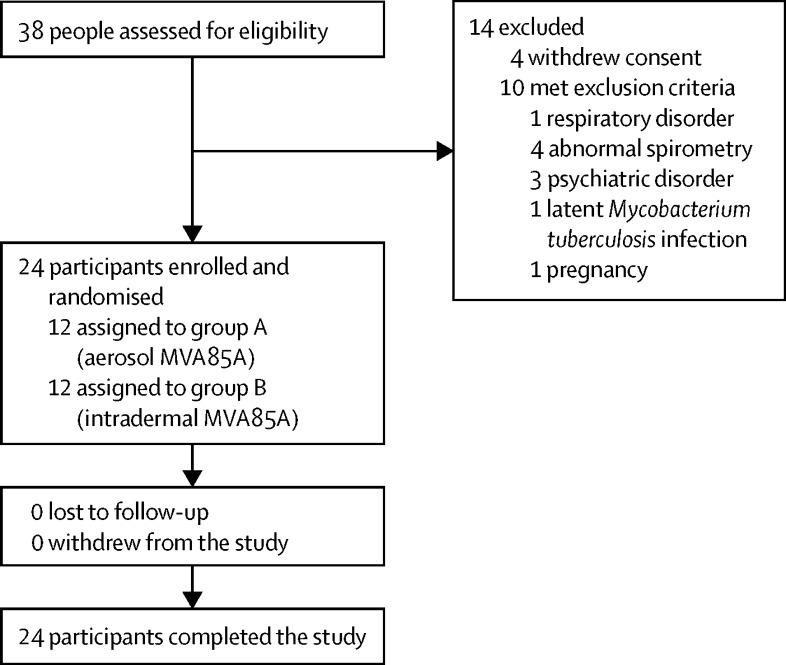
Trial profile

Between Nov 10, 2011, and Nov 1, 2012, 38 healthy adults in the UK were screened for inclusion in the trial, and 14 were excluded (figure 1). Therefore, 24 adults were judged eligible for inclusion. The baseline characteristics of the study participants did not differ significantly between treatment groups (table 1).

**Table 1:** Baseline characteristics

		**Group A: aerosol MVA85A (n=12)**	**Group B: intradermal MVA85A (n=12)**
Women		7 (58%)	7 (58%)
Age (years)		21·5 (20–49)	22·0 (20–26)
Time since BCG vaccination (years)		9·0 (1–49)	9·0 (5–23)
Non-smokers		12 (100%)	12 (100%)
BMI (kg/m^2^)		22·6 (19·8–37·7)	21·3 (18·8–29·4)
Spirometry			
	% predicted FEV_1_	99% (80–110)	91% (85–110)
	% predicted FVC	100% (83–108)	91% (82–103)
Median baseline % S_p_O_2_		98% (97–99)	98% (97–99)
Continent of birth			
	Europe	11 (92%)	12 (100%)
	Africa	1 (8%)	0
	Asia	0	0

BMI=body-mass index. FEV_1_=forced expiratory volume in 1 s. FVC=forced vital capacity. S_p_O_2_=peripheral oxygen saturation. Data are n (%) or median (range), unless otherwise indicated.

**Figure 2:**
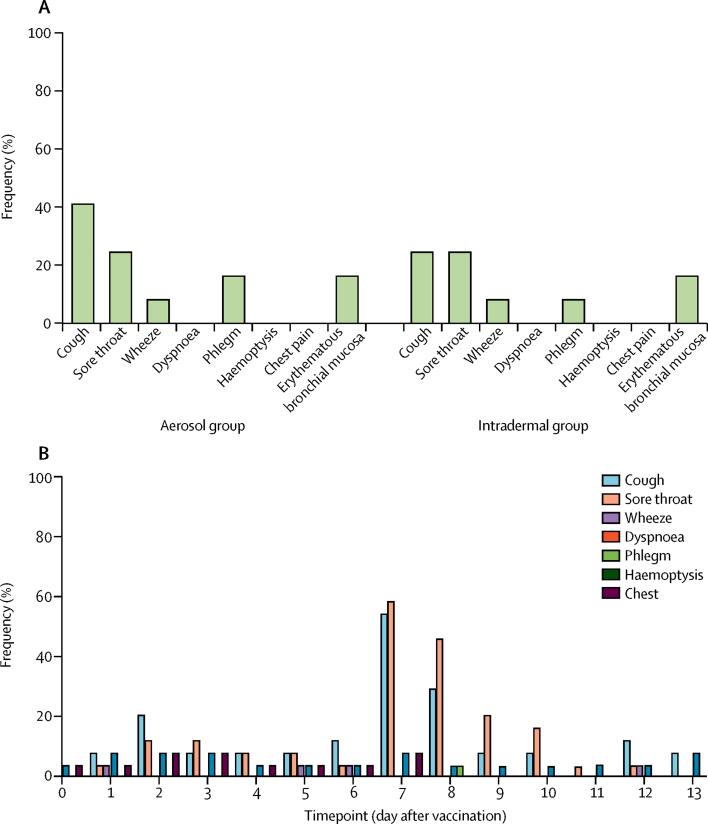
Respiratory and systemic adverse events after bronchoscopy and bronchoalveolar lavage (A) Frequency of mild respiratory adverse events in each group during the first 7 days after vaccination. All events were mild and did not differ significantly between the groups. (B) Frequency of respiratory adverse events on each of the 14 days after vaccination for both groups combined. Day 7 is the day of bronchoscopy. As expected, bronchoscopy and bronchoalveolar lavage was associated with non-sustained respiratory adverse events, especially cough and sore throat, in some participants.

The number of respiratory adverse events did not differ between participants receiving aerosol saline placebo and those receiving aerosol MVA85A (median 1·0 events per person for saline placebo *vs* 0·5 for MVA85A; p=0·76). Respiratory adverse events were rare and mild in both groups (figure 2). The most frequent expected (and mild) respiratory adverse events after bronchoscopy were cough and sore throat (figure 2).

**Figure 3:**
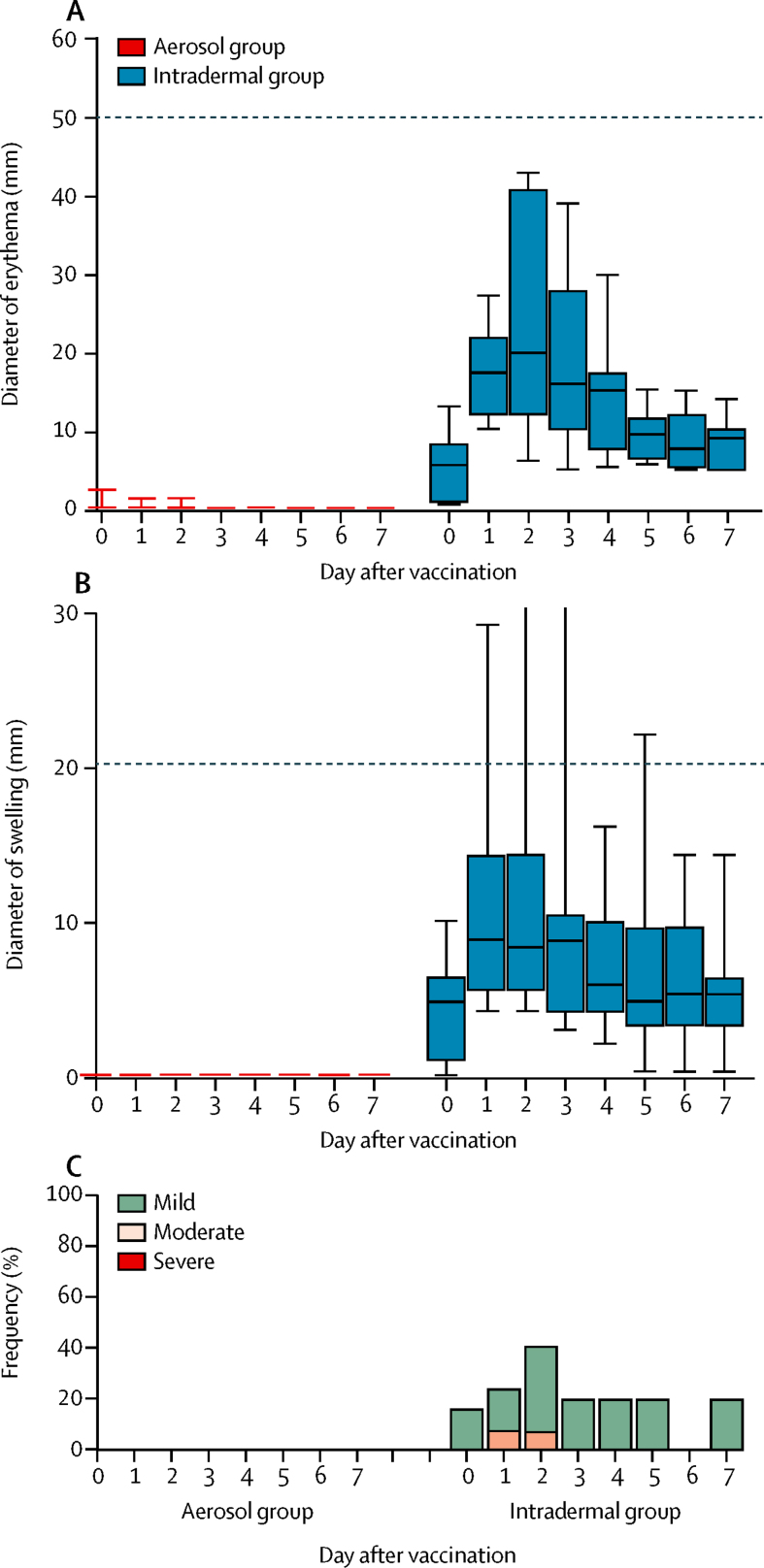
Local and systemic adverse events associated with aerosol and intradermal MVA85A vaccination Box and whisker plots showing the diameter (mm) of injection-site erythema (A) and swelling (B) in participants in each group for 7 days after vaccination. The dashed line on each graph represents the threshold between mild and moderate grading. Local reactions were negligible in the aerosol group compared with the intradermal group. (C) Frequency and severity of injection-site pain in each of the first 7 days after vaccination with either aerosol or intradermal MVA85A. Participants given aerosol MVA85A received a concurrent intradermal injection of saline, which was non-reactogenic.

Intradermal MVA85A was associated with an expected mild local injection-site reaction (injection-site erythema, swelling, or other mild local skin reaction; figure 3).^11^ Intradermal saline was non-reactogenic.

Systemic adverse events did not differ significantly between the groups (table 2). Three participants in each group had no vaccine-related systemic adverse events. In the other participants, mild fatigue (11/24 [46%]) and mild-to-moderate headache (10/24 [42%]) were the most common symptoms (mostly mild, but two participants had moderate headache in group A). One person in each group reported feverishness after vaccination. A documented mild fever (37·7°C) occurred in one intradermally vaccinated participant, on the evening after vaccination.

**Table 2:** Frequency and severity of expected systemic adverse events in each group in the first 7 days after vaccination

	**Group A: aerosol MVA85A (n=12)**	**Group B: intradermal MVA85A (n=12)**
Fever	0	1 (8%)
Feverish	1 (8%)	1 (8%)
Arthralgia	0	1 (8%)
Myalgia	0	2 (17%)
Malaise	2 (17%)	2 (17%)
Fatigue	5 (42%)	6 (50%)
Headache	5 (42%)	5 (42%)
Nausea and vomiting	1 (8%)	0

Most expected systemic adverse events were mild (with the exception of two cases of moderate headache in the aerosol group) and did not differ clinically between the groups. For simplicity, we report the adverse events of the 12 participants in each group collectively, even though the first two participants (both in group A) received a tenfold higher dose of vaccine than did the other 22 participants.

The first participant vaccinated by the aerosol route, who received the higher dose of MVA85A (1 × 10^8^ pfu), reported a mild vesicular skin rash on the right side of her face that appeared 11 days after vaccination. This rash was diagnosed as shingles (varicella zoster virus) in the right C2 dermatome by her family doctor and confirmed by viral PCR. The patient was given oral aciclovir and simple analgesics and recovered completely in 10 days. Shingles was not detected in any other participant.

No clinically significant abnormalities of laboratory tests at 7 days or 84 days after vaccination were recorded. No serious adverse events occurred in the trial.

S_p_O_2_ was normal and stable (mean S_p_O_2_ >97%) throughout the trial in all but one participant (in the intradermal MVA85A group) who developed hypersalivation, profuse coughing, and transient desaturation to S_p_O_2_ 88%, before bronchoscopic cannulation of the vocal cords. The participant was given oxygen and recovered rapidly in about 60 s; bronchoscopy then proceeded uneventfully. This event was judged to be unrelated to vaccination.

**Figure 4:**
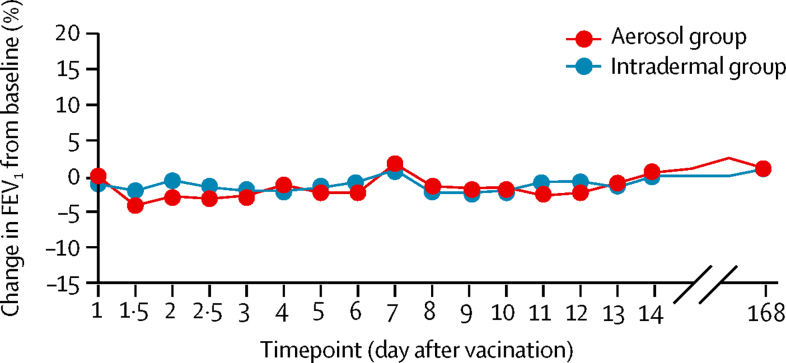
Mean percentage change in FEV_1_ from baseline at each timepoint for both treatment groups FEV_1_=forced expiratory volume in 1 s.

Treatment had no clinically significant effect on FEV_1_ in either group, from immediately after vaccination (at 10 min and 60 min) and throughout follow-up (figure 4). Four people were judged to have mildly erythematous mucosa at bronchoscopy, two of whom had received aerosol MVA85A and two intradermal MVA85A (and aerosol saline). The bronchial mucosa of the other 20 participants was normal. Bronchoalveolar lavage was obtained successfully from all participants with a median return volume of 46 mL from a 100 mL lavage (equating to a median yield of 5·4 × 10^6^ lymphocytes).

For the secondary outcome of immunogenicity, both aerosol and intradermal MVA85A (1 × 10^7^ pfu) induced significant systemic Ag85A-specific responses, as measured by ex-vivo interferon-γ ELISpot assay (spot-forming cells per 1 × 10^6^ peripheral blood mononuclear cells; appendix). AUC analysis did not differ between the groups (p=0·9212). Both routes of vaccination with 1 × 10^7^ pfu MVA85A also induced MVA CD4 and CD8 T-cell responses (appendix). The MVA CD4 T-cell response was higher in the aerosol group than in the intradermal group (p=0·0358), but the size of the MVA CD8 T-cell response did not differ significantly between the two groups (p=0·5319).

2·5 million to 9 million cells were recovered from bronchoalveolar lavage, which was independent of the vaccine administration route (data not shown).

Both intradermal and aerosol MVA85A (1 × 10^7^ pfu) induced CD4 T cell interferon γ, TNFα, interleukin 2, and interleukin 17 in the bronchoalveolar lavage. The aerosol administration route induced higher numbers of some Ag85A-specific CD4 cell cytokines (appendix).

In the whole blood, interferon-γ-producing CD4 T cells increased significantly after vaccination (appendix), and remained significantly increased compared with baseline for up to 4 weeks in the aerosol group and up to 24 weeks in the intradermal group. The numbers of CD4 T cells making TNFα, interleukin 2, and interleukin 17 increased after vaccination in both groups (appendix). Both routes of vaccination induced CD4 T cells that produced several cytokines (appendix).

Ag85A-specific interferon-γ-producing CD8 T cells were detectable in the bronchoalveolar lavage in both groups (appendix). No other cytokines were detected in the Ag85A-specific CD8 T-cell population (data not shown).

Aerosol vaccination did not induce increased non-specific lung cytokine responses compared with intradermal vaccination (data not shown).

Anti-MVA IgG titres were significantly higher in the low-dose intradermal group than in the low-dose aerosol group at days 14, 28, 84, and 168 after vaccination (appendix; p<0·0001). Anti-MVA IgA titres were significantly higher in the low-dose intradermal group than in the aerosol group from day 14 after vaccination (appendix; p=0·0003). The amounts of anti-MVA IgM did not differ significantly between the two groups (appendix).

No significant effect on Ag85A IgG, IgA, and IgM responses was detected in either group (appendix). No antibodies were detectable in bronchoalveolar lavage fluid (data not shown).

In the two participants who received the high dose (1 × 10^8^ pfu) of aerosol MVA85A, blood and bronchoalveolar lavage ex-vivo interferon-γ ELISpot responses exceeded the limit of detection using 3 × 10^5^ cells per well; therefore, data from 1 × 10^5^ cells per well are presented (table 3). One participant had high anti-MVA CD4 and CD8 T-cell interferon-γ responses at 1 week after vaccination. High numbers of cells making Ag85A-specific cytokines were detected in the bronchoalveolar lavage from both participants. Most interferon-γ-producing CD4 T cells also produced TNFα and interleukin 17. Interferon γ from CD8 T cells was also detected in these samples. Frequencies of whole blood CD4 T cells producing interferon γ, TNFα, interleukin 2, and interleukin 17 were also high in these two participants (table 3). Anti-MVA antibody responses were similar to those detected in the low-dose aerosol group (table 3).

**Table 3:** Immune responses at 1 week after vaccination in two participants who received high-dose aerosol MVA85A compared with median responses for participants given lower dose

	**High-dose (1 × 10^8^ pfu) participant TB026-301**	**High-dose (1 × 10^8^ pfu) participant TB026-303**	**Median (IQR) response for low-dose (1 × 10^7^ pfu) aerosol MVA85A**	**Median (IQR) response for low-dose (1 × 10^7^ pfu) intradermal MVA85A**
**Ex-vivo PBMC interferon-γ ELISpot responses (spot-forming cells/1 × 10**^6^**PBMCs)**				
PBMC 85A interferon γ (1 × 10^5^ cells/well)	4985	5000	1965 (490–3659)	1010 (681–1490)
PBMC MVA CD4 interferon γ (3 × 10^5^ cells/well)	212	10	16 (5–111)	10 (1–21)
PBMC MVA CD8 interferon γ (3 × 10^5^ cells/well)	817	30	2·5 (1–46·5)	13·5 (1·3–24·3)
**Intracellular cytokines in BAL and WB (% of T cells producing cytokines)**				
BAL CD4 interferon γ	17·3%	21·2%	2·0% (1·1–5·8)	0·7% (0·3–1·2)
BAL CD4 TNFα	10·5%	19·3%	2·3% (0·9–5·2)	0·6% (0·1–1·9)
BAL CD4 interleukin 17	3·2%	6·8%	0·6% (0·3–1·4)	0·2% (0·1–0·3)
BAL CD8 interferon γ	0·7%	2·5%	0·1% (0·1–0·6)	0·1% (0·03–0·3)
WB CD4 interferon γ	1·4%	0·7%	0·3% (0·1–0·6)	0·1% (0·1–0·2)
WB CD4 TNFα	1·3 %	0·6%	0·2% (0·1–0·4)	0·1% (0·1–0·2)
WB CD4 interleukin 2	1·1%	0·6%	0·1% (0·1–0·3)	0·1% (0·04–0·1)
WB CD4 interleukin 17	0·1%	0·1%	<0·1% (0·01–0·1)	<0·1% (0·0–0·02)
**Antibody responses in serum samples at week 1 (optical density values)**				
MVA IgG	0·5	0·4	0·6 (0·4–1·2)	0·6 (0·5–0·9)
MVA IgA	0·2	0·3	0·4 (0·3–0·5)	0·5 (0·4–0·6)
MVA IgM	1·4	1·1	0·9 (0·7–1·2)	0·9 (0·6–1·3)

All presented data are background subtracted. pfu=plaque-forming units. PMBC=peripheral blood mononuclear cells. BAL=bronchoalveolar lavage. WB=whole blood. TNF=tumour necrosis factor.

## Discussion

Our results show that aerosol vaccination with MVA85A is feasible, safe, and can induce mycobacteria-specific mucosal and systemic cellular immune responses.

Respiratory adverse events after aerosol delivery were rare and did not differ between the two groups, which suggests that the adverse events are caused by the nebulised delivery technique rather than by MVA85A itself. Bronchoscopic airway examination, FEV_1_, FVC, and S_p_O_2_ were within normal limits, providing further reassuring evidence for the absence of harm to the airways. Systemic reactogenicity of MVA85A administered by both routes was mild, which is consistent with previous trials.

Although an isolated case of shingles occurred in one participant 11 days after aerosol administration of MVA85A, no previous vaccine-related cases of shingles have been reported in more than 2500 recipients of intradermal MVA85A (H McShane, unpublished). Shingles is quite common in the general population and has been reported to occur before 45 years of age in 8·6% of healthy men and 10·5% of healthy women.^15^ In a recent US phase 1 trial of a novel live recombinant BCG tuberculosis candidate vaccine, two of eight participants developed shingles within 2–3 months of vaccination.^16^

The aerosol route of immunisation induced potent antigen-specific T-cell responses in the bronchoalveolar lavage and in the systemic circulation. Compared with the intradermal vaccination route, the aerosol route induced equally strong systemic responses and significantly stronger bronchoalveolar lavage responses. The cytokines measured—interferon γ, TNFα, interleukin 2, and interleukin 17—are important for the control of mycobacterial infection.^17^, ^18^, ^19^, ^20^ The intradermal route also induced lung CD4 T-cell responses, although these were of a smaller size than those induced by the aerosol route. In non-human primates, systemic administration of MVA85A induced mucosal responses at a lower level than recorded in people;^8^ however, in mice, systemic delivery of a recombinant adenoviral vector does not induce mucosal responses.^21^

CD8 T cells are thought important in defence against tuberculosis.^22^, ^23^ We detected antigen-specific CD8 T cells in bronchoalveolar lavage in both groups. Mucosal CD8 T cells are inducible by mucosal vaccination with adenovirus-vectored Ag85A and are crucial for protection against tuberculosis.^21^

Although we noted a rise in the numbers of lung cells making cytokines in the aerosol group, this increase was not accompanied by an increase in the total number of lymphocytes recruited to the lungs. This finding contradicts a previous study reporting a threefold increase in total bronchoalveolar lavage cells in purified protein derivative-reactive people challenged mucosally with purified protein derivative.^24^ This discrepancy might be attributable to the nature of the antigen used, which was Ag85A in our study. An increase in influx of lymphocytes to the lungs was mediated by interferon-γ-inducible and interleukin-17-inducible CXCR3;^20^, ^25^ in the present study we did not assess the expression of this chemokine.

Pre-existing immunity to the vector might reduce insert-induced immunity.^26^ Here, we show that systemic administration of the vaccine induced systemic antivector humoral immune responses—a phenomenon that is overcome by aerosol vaccination. In this study, we could not detect antivector or anti-insert antibodies in bronchoalveolar fluid from any participants, possibly because the fluid is highly diluted. A vaccination route that does not induce immunity to the vector, which could interfere with insert-induced responses, is an attractive approach for vaccines that need repeat administration.

The two participants who received high-dose MVA85A had higher systemic and mucosal Ag85A responses than did those who received the lower dose. Because the two participants who received the high dose were randomly allocated to the aerosol group, whether or not a similarly high intradermal dose would lead to an increase in cellular mucosal responses is unclear. One of the two participants who had the high dose had a strong anti-MVA interferon-γ response (both CD4 and CD8 T cells).

MVA85A has been given intradermally in all previous trials in human beings, including the recent efficacy trial in tuberculosis-exposed BCG-vaccinated infants, in which immunogenicity was modest and no significant enhanced efficacy was recorded.^27^ Direct delivery of the vaccine to the lungs might improve immunogenicity and efficacy. Nebulisation of BCG provided superior protection against *M tuberculosis* challenge compared with that conferred by intradermal vaccination in non-human primates.^28^ The immunological basis for this enhanced protection is not clear. Preferential circulation of memory lymphocytes back to the tissues in which they first encountered antigen might underlie the limited efficacy of the intradermally administered BCG.^29^ Delivery of a tuberculosis vaccine by aerosol to people is not a novel approach. Nebuliser delivery of BCG to people induces systemic immune responses as measured by a tuberculin skin test.^30^

In this phase 1 trial, we describe the first aerosol administration of a candidate tuberculosis vaccine to human beings and show that the vaccine is safe and strongly immunogenic (panel). Further studies are needed to fully characterise the mucosal and systemic immune response to both insert and vector. This work is of relevance for all respiratory pathogens for which a mucosal immune response is desirable.

Panel **Research in context**

**Systematic review**

We searched PubMed using the terms “TB vaccine”, “aerosol”, and “clinical trial”, with no restrictions on our search dates or language. We found no reported trials of this kind, and our trial is the first report of a clinical trial in which a candidate tuberculosis vaccine has been delivered by aerosol to human participants. The only other published study used BCG, the only licensed vaccine against tuberculosis, delivered by nebuliser in a sealed room to a group of healthy human participants.^29^ In this study, two different nebulisation devices were used, and a sealed room and a small chamber were also compared. Rates of tuberculin skin test conversion were assessed in guinea pigs and healthy human participants.

**Interpretation**

Our trial shows that aerosol vaccination with MVA85A is feasible, safe, and can induce potent mycobacteria-specific mucosal and systemic cellular immune responses. Further studies are needed to fully characterise the mucosal and systemic immune response to both insert and vector. This work is of relevance for all respiratory pathogens against which a mucosal immune response is desirable.
