# Supplementary material for: Safety and immunogenicity of a candidate tuberculosis vaccine MVA85A delivered by aerosol in BCG-vaccinated healthy adults: a phase 1, double-blind, randomised controlled trial
Source: Lancet Infect Dis. 2014 Aug 20;14(10):939–46. doi: 10.1016/S1473-3099(14)70845-X (PMC4178237; doi:10.1016/S1473-3099(14)70845-X)
Supplement: Supplementary appendix [file mmc1.pdf]

THE LANCET Infectious Diseases

Supplementary webappendix

This webappendix formed part of the original submission and has been peer reviewed. We post it as supplied by the authors.

Supplement to: Satti I, Meyer J, Harris SA, et al. Safety and immunogenicity of a candidate tuberculosis vaccine MVA85A delivered by aerosol in BCG-vaccinated healthy adults: a phase 1, double-blind, randomised controlled trial. *Lancet Infect Dis* 2014; published online Aug 21. http://dx.doi.org/10.1016/S1473-3099(14)70845-X.

Web appendix

Figure 1

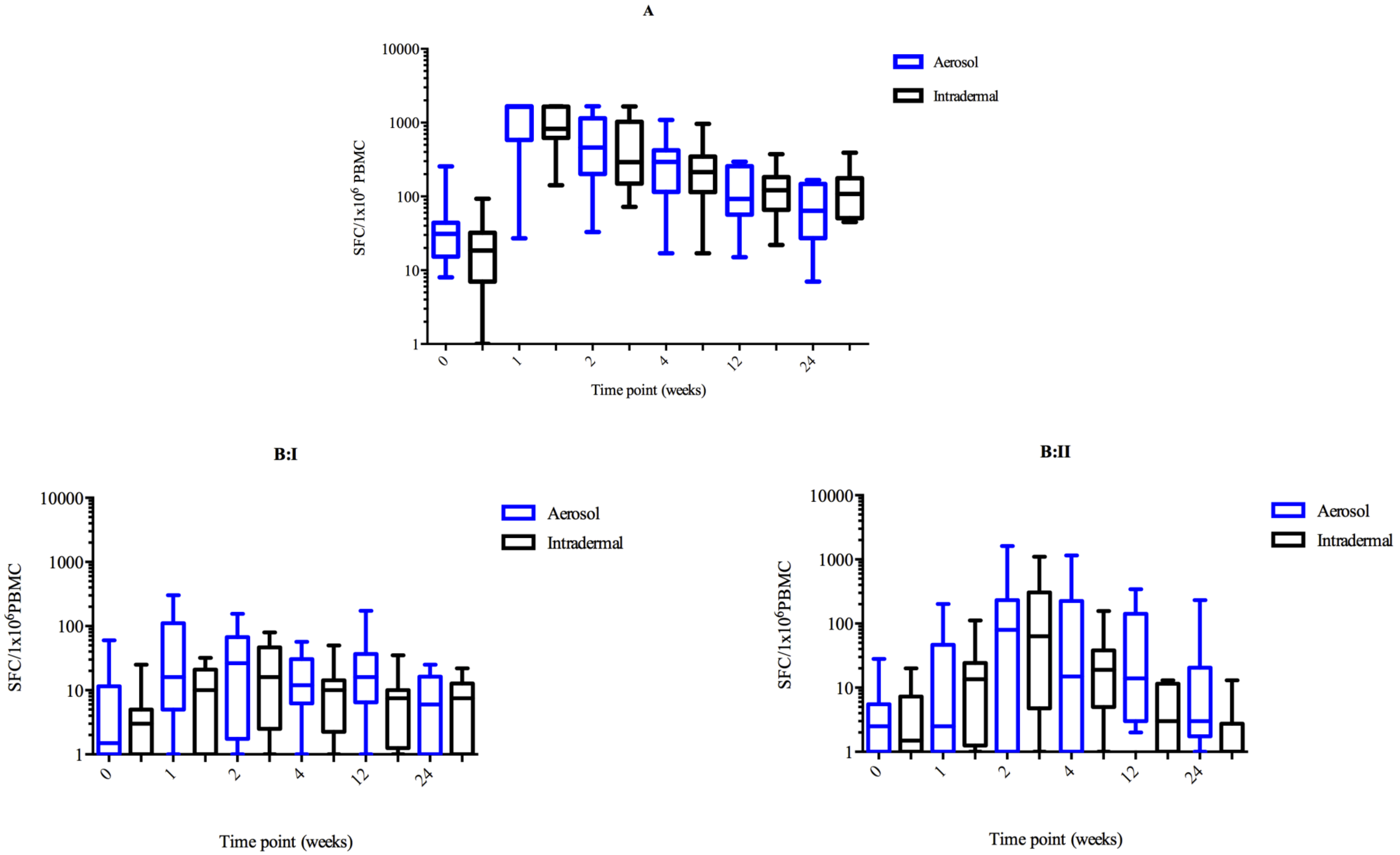

Figure 1: *Ex-vivo* IFNγ ELISpot responses.

(A) Shows IFNγ to Ag85A peptides in the aerosol and intradermal groups. IFNγ increased significantly for 12 and 24 weeks after aerosol and intradermal vaccination respectively. No significant difference was detected in the IFNγ between the groups (p=0·9212).

(B) Anti-vector responses: IFNγ responses were assessed to CD4+ and CD8+ T cell epitopes present in Vaccinia and MVA.

   I. MVA CD4+ T cell IFNγ: Significant increase in response followed aerosol vaccination at 1 and 2 weeks, p= 0·0469 and 0·0293 respectively. The intradermal response increased significantly at week 2 post-vaccination (p=0·0352). Mann-Whitney test for AUC shows that the aerosol group had significantly higher MVA CD4+ T cell IFNγ response than the intradermal group (p=0·0358).

   II. MVA CD8+ T cell IFNγ: Responses increased significantly in the aerosol group at 2, 4, and 12 weeks post-vaccination (p<0·05), whereas the intradermal group MVA-CD8 response increased significantly from the first week post-vaccination up to week 4 (p<0·05). Levels of MVA CD8+ T cell IFNγ were not significantly different in the two groups (p=0·5319).

Box and whisker plots show median, interquartile range, and minimum and maximum values. Presented results are antigen-specific with unstimulated background responses subtracted.

Figure 2

A

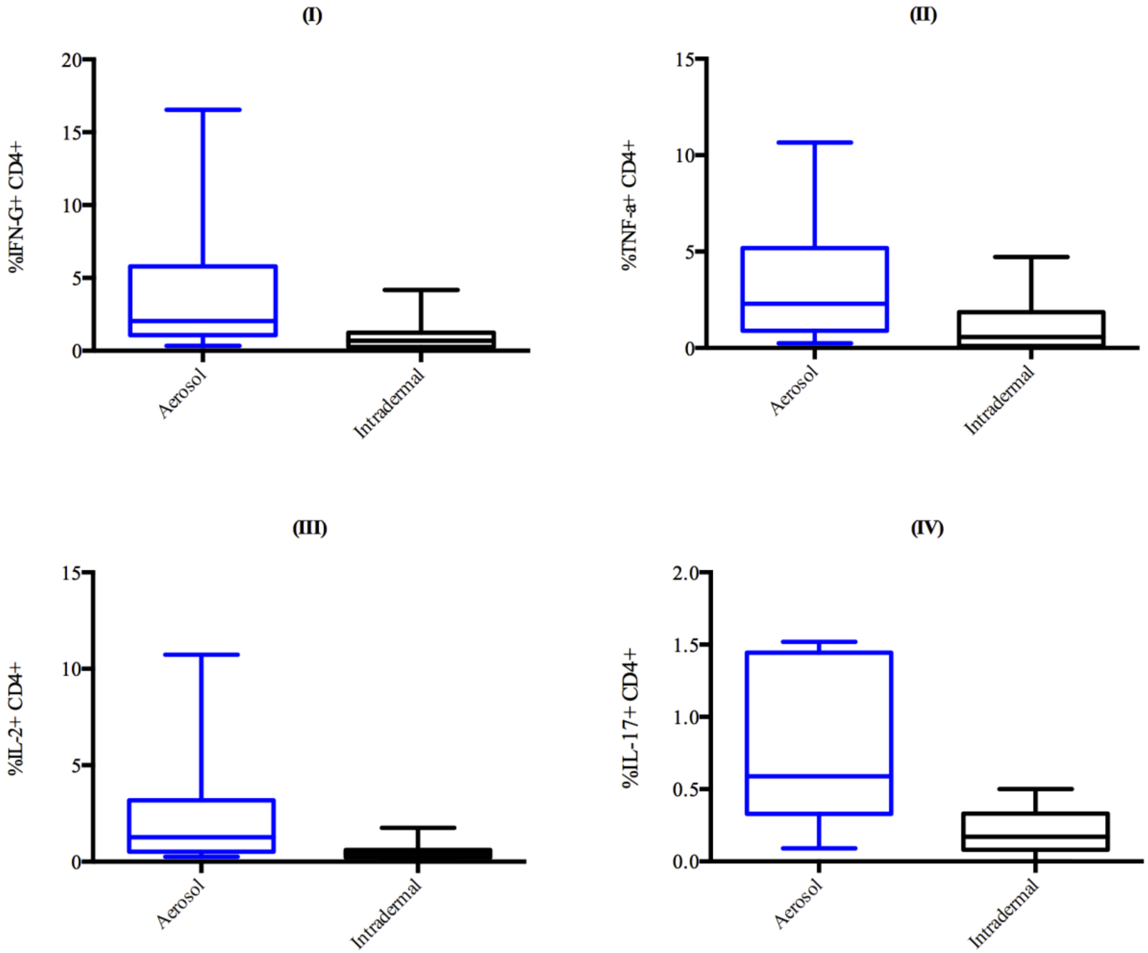
(I)
%IFN-G+ CD4+
20
15
10
5
0
Aerosol
Intradermal
(II)
%TNF-a+ CD4+
15
10
5
0
Aerosol
Intradermal
(III)
%IL-2+ CD4+
15
10
5
0
Aerosol
Intradermal
(IV)
%IL-17+ CD4+
2.0
1.5
1.0
0.5
0.0
Aerosol
Intradermal

B

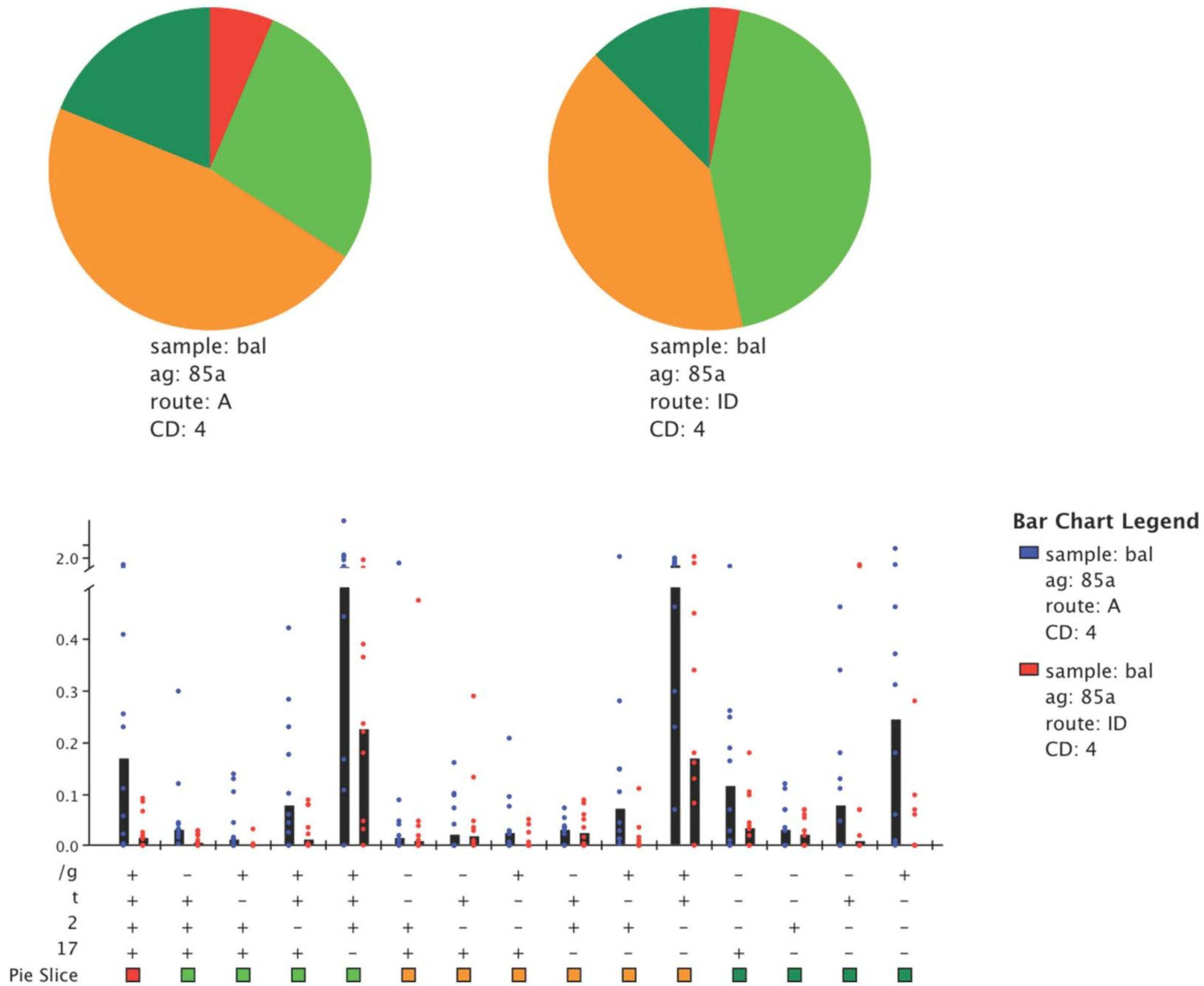
sample: bal
ag: 85a
route: A
CD: 4
sample: bal
ag: 85a
route: ID
CD: 4
2.0
0.4
0.3
0.2
0.1
0.0
/g
t
2
17
Pie Slice
Bar Chart Legend
sample: bal
ag: 85a
route: A
CD: 4
sample: bal
ag: 85a
route: ID
CD: 4

C

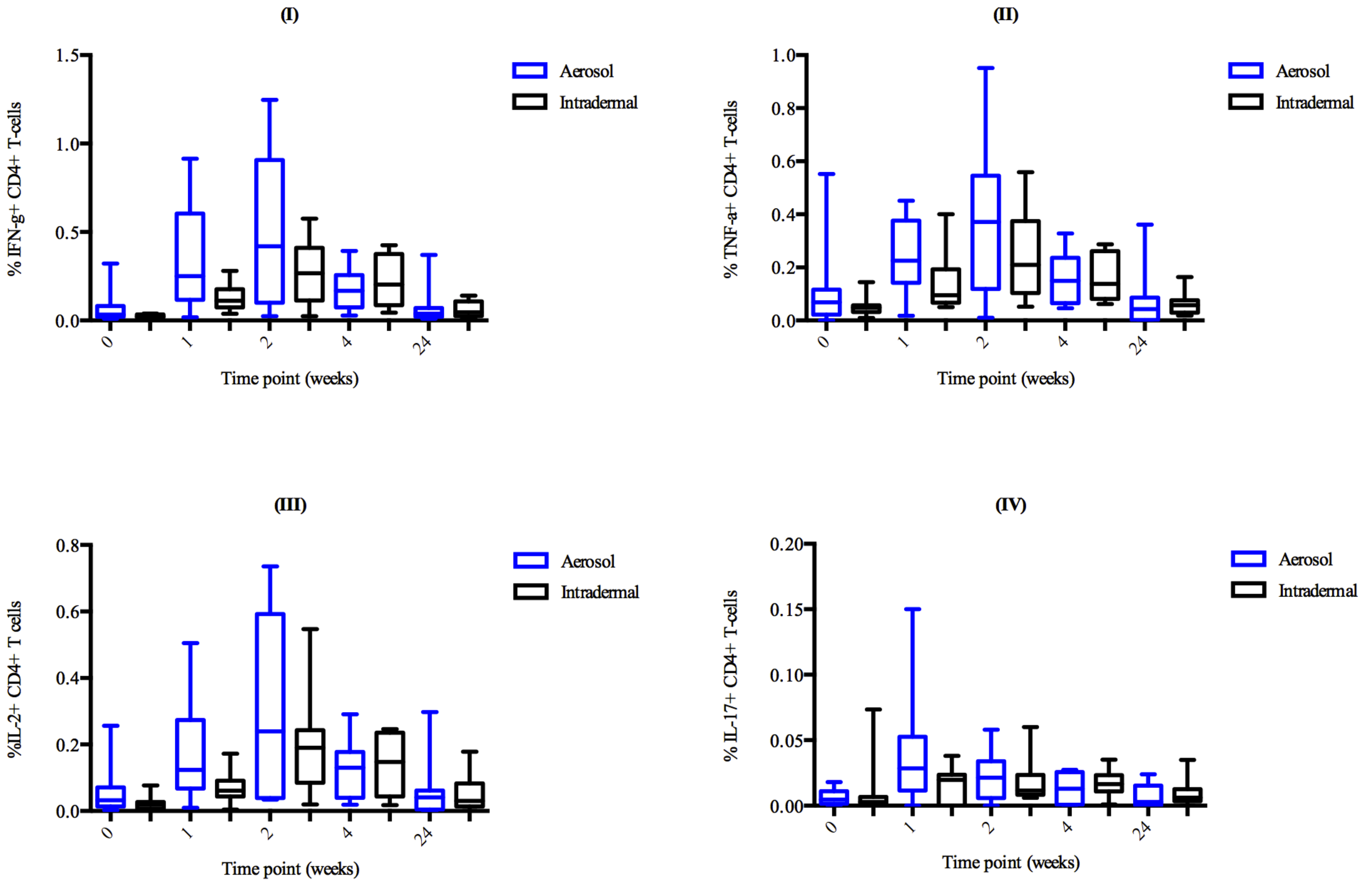
(I)
1.5
1.0
0.5
0.0
% IFN-g+ CD4+ T-cells
0
1
2
4
24
Time point (weeks)
Aerosol
Intradermal
(II)
1.0
0.8
0.6
0.4
0.2
0.0
% TNF-a+ CD4+ T-cells
0
1
2
4
24
Time point (weeks)
Aerosol
Intradermal
(III)
0.8
0.6
0.4
0.2
0.0
%IL-2+ CD4+ T cells
0
1
2
4
24
Time point (weeks)
Aerosol
Intradermal
(IV)
0.20
0.15
0.10
0.05
0.00
% IL-17+ CD4+ T-cells
0
1
2
4
24
Time point (weeks)
Aerosol
Intradermal

D

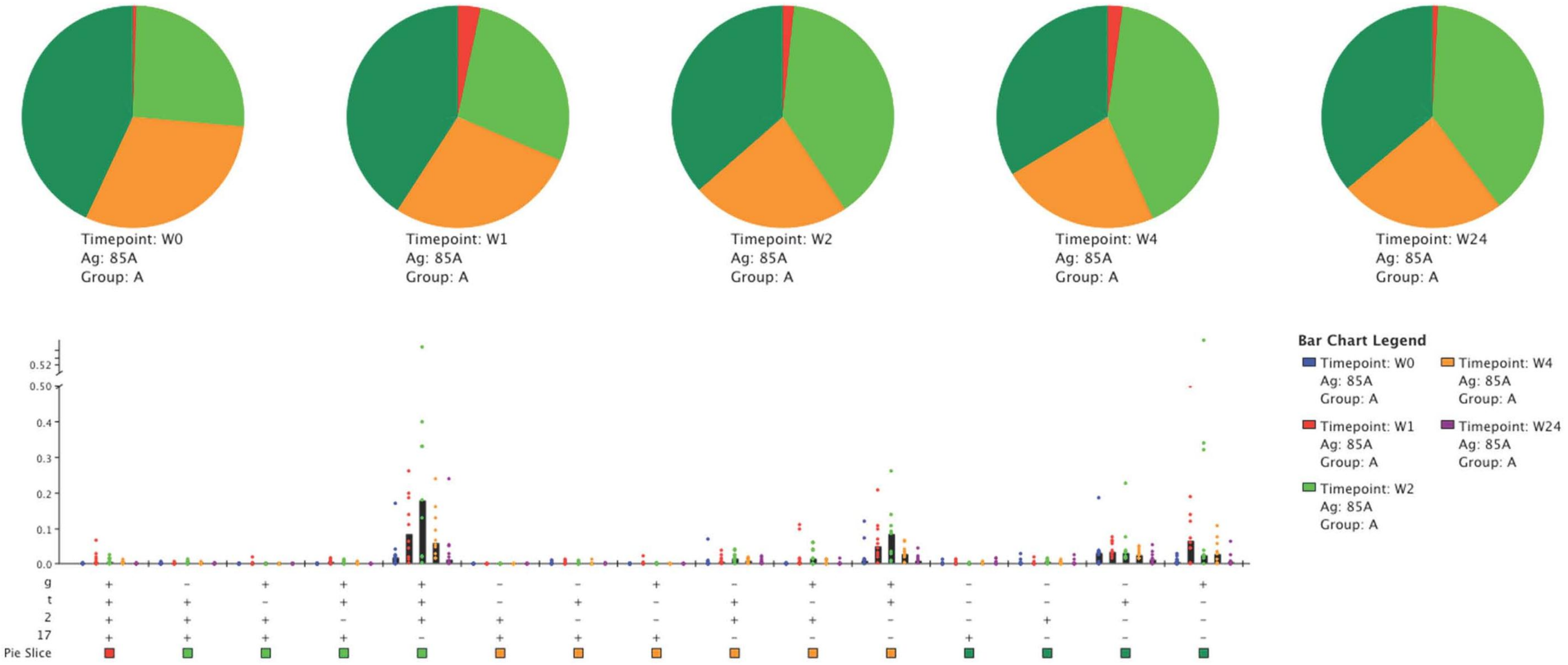
Timepoint: W0
Ag: 85A
Group: A
Timepoint: W1
Ag: 85A
Group: A
Timepoint: W2
Ag: 85A
Group: A
Timepoint: W4
Ag: 85A
Group: A
Timepoint: W24
Ag: 85A
Group: A
Bar Chart Legend
Timepoint: W0 Ag: 85A Group: A
Timepoint: W1 Ag: 85A Group: A
Timepoint: W2 Ag: 85A Group: A
Timepoint: W4 Ag: 85A Group: A
Timepoint: W24 Ag: 85A Group: A
0.52
0.50
0.4
0.3
0.2
0.1
0.0
g
t
2
17
Pie Slice

D continued

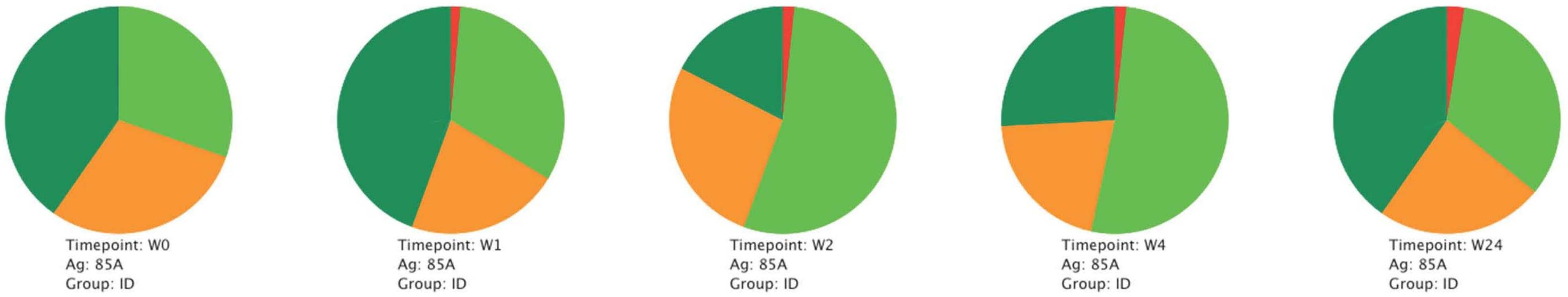
Timepoint: W0
Ag: 85A
Group: ID
Timepoint: W1
Ag: 85A
Group: ID
Timepoint: W2
Ag: 85A
Group: ID
Timepoint: W4
Ag: 85A
Group: ID
Timepoint: W24
Ag: 85A
Group: ID

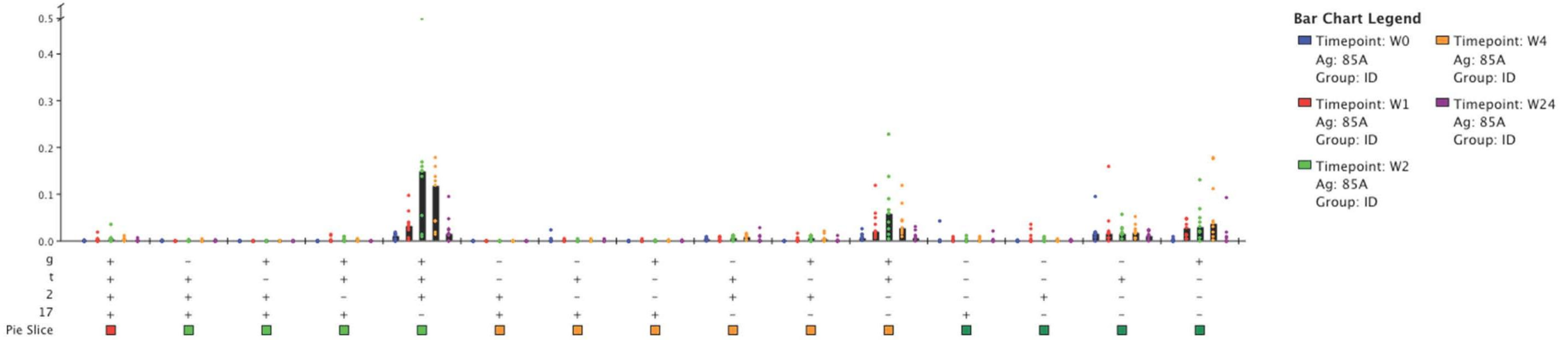
0.5
0.4
0.3
0.2
0.1
0.0
g
t
2
17
Pie Slice
Bar Chart Legend
Timepoint: W0 Ag: 85A Group: ID
Timepoint: W1 Ag: 85A Group: ID
Timepoint: W2 Ag: 85A Group: ID
Timepoint: W4 Ag: 85A Group: ID
Timepoint: W24 Ag: 85A Group: ID

E

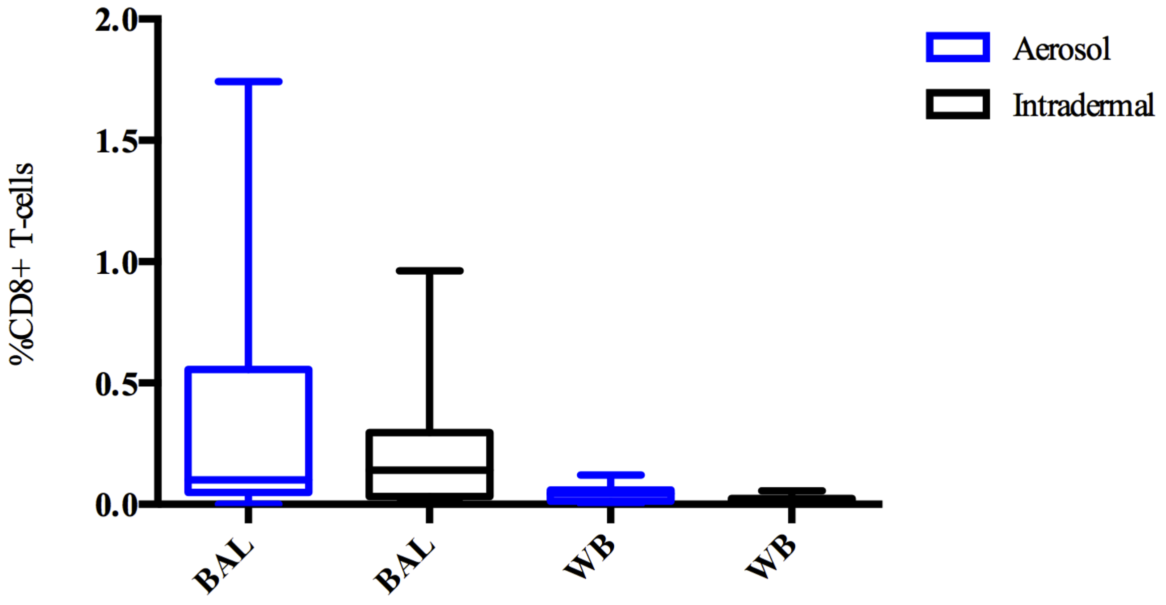

Figure 2 : Ag85A-specific intracellular cytokine responses.

(A) BAL CD4+ T cell cytokines measured after an overnight stimulation of cells with Ag85A single peptide pool at 1 week after MVA85A vaccination: Lung CD4+T cell IFNγ, TNFα, IL-2, and IL-17 were higher in the aerosol group with p values =0·033, 0·05, 0·04, and 0·015 respectively (Figure A:I-IV).

(B) Polyfunctional BAL CD4+ T cell responses: Both routes of vaccination induced production of multiple mycobacteria-specific cytokines by CD4+ T cells in the lungs. Frequency of cells simultaneously making IFNγ, TNFα, IL-2, and IL-17, double positive IFNγ/IL-2, or IFNγ/IL-17 were significantly higher in the aerosol group ($p<0·05$).

(C) WB ICS cytokines: Systemic intracellular cytokine response measured in WB samples collected over a period of 24 weeks post-vaccination:

(I) Intracellular WB CD4+ T cell IFNγ significantly increased in both groups post-vaccination up to 24 week in the intradermal group and up to 4 weeks in the aerosol group ($p<0·05$).

(II) WB CD4+ T cell TNFα significantly increased up to 4 and 2 weeks following MVA85A intradermal and aerosol vaccination respectively ($p<0·05$).

(III) WB CD4+ T cell IL-2 increased significantly up to 24 weeks after intradermal vaccination and for the first 2 weeks after aerosolised MVA85A ($p<0·05$).

(IV) WB CD4+ T cell IL-17 significantly increased for the first 2 weeks following aerosol vaccination and peaked at the first week after intradermal administration of MVA85A ($p<0·05$).

Mann-Whitney test for AUC shows no significant difference in frequencies of cells making any of the studied cytokines between the two vaccination groups.

(D) Multiple WB cytokines following MVA85A vaccination: Frequency of CD4+T cells simultaneously producing IFNγ/TNFα/IL-2 was higher in the aerosol group, but this is not significant, the same is for IFNγ/TNFα, IFNγ/IL-2, and IFNγ/IL-17 double positive cells.

(E) Higher numbers of CD8+ T cells produced IFNγ in the BAL as compared to WB; this was not affected by route of vaccination.

Box and whisker plots show median, interquartile range, and minimum and maximum values.

Figure 3

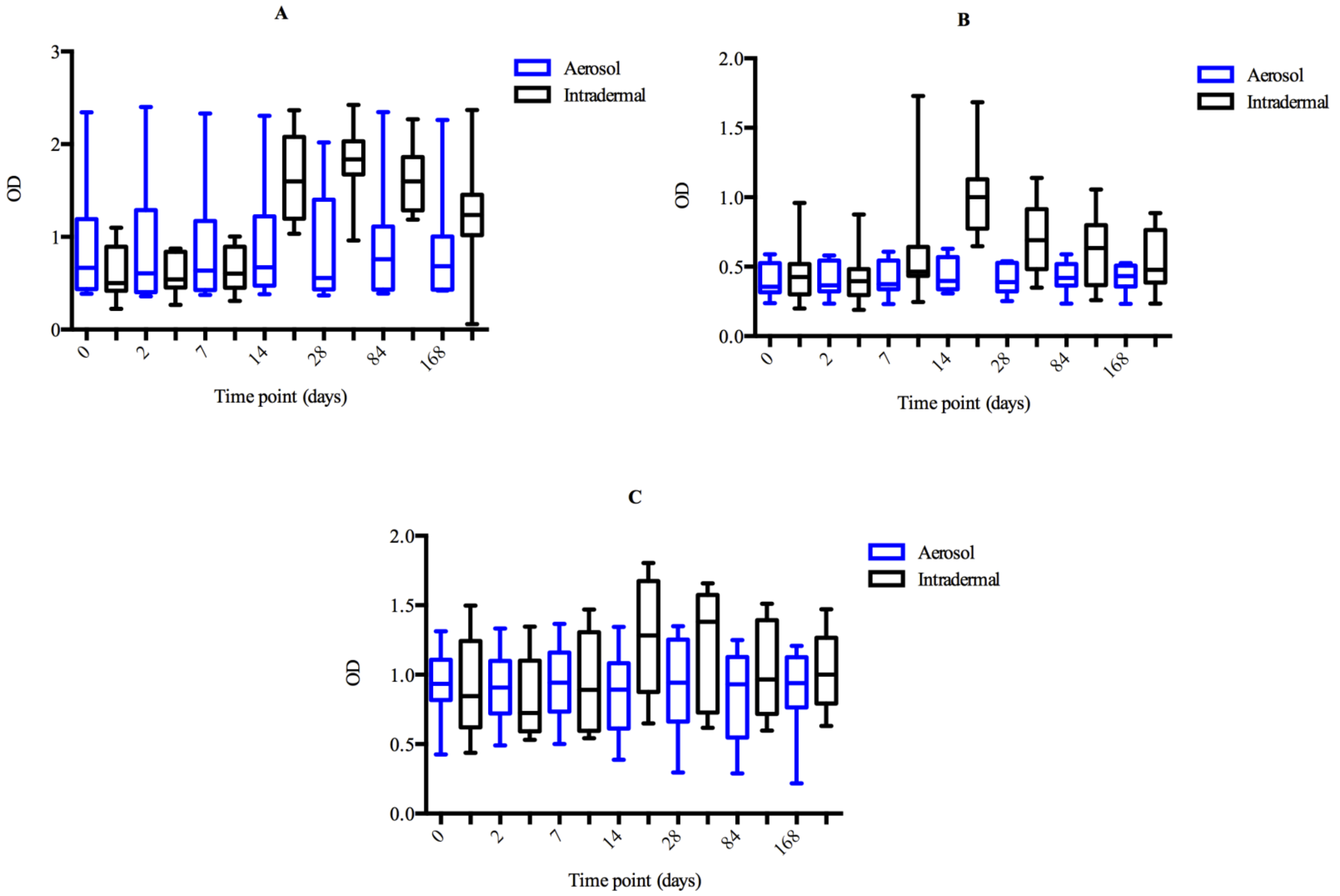

Figure 3: Systemic anti-vector antibody responses in serum samples.

(A) Intradermal vaccination with MVA85A induced significantly high anti-MVA serum IgG at days 14, 28, 84, and 168 post-vaccination. The intradermal group IgG was significantly higher than the aerosol group response ($p<0{\cdot}0001$).

(B) Serum anti-vector IgA peaked at D14 in the intradermal group and remained significantly higher up to the last follow up time point, no significant differences were observed in the aerosol group. The intradermal group IgA response was significantly higher than the aerosol group ($p=0{\cdot}0003$).

(C) IgM to MVA increased significantly in the intradermal vaccinated group at days 14, 28, and 84 post-vaccination, while levels remained unchanged in the aerosol group. However, these IgM responses, detected in the intradermal group were not significantly different to the aerosol group's response ($p=0{\cdot}079$).

Box and whisker plots show median, interquartile range, and minimum and maximum values.

Appendix table 1: Serum-Ag85A antibody responses in volunteers vaccinated with aerosol or intradermal MVA85A ($1x10^7$pfu):

Appendix table 1: Ag85A serum antibody responses (OD values)

A: Aerosol $1x10^7$ MVA85A

	Day 0	Day 2	Day 7	Day 14	Day28	Day84	Day168
IgA	0.02925	0.02575	0.02775	0.0245	0.0275	0.0415	0.03
IgG	0.1435	0.17375	0.15575	0.16	0.151	0.1585	0.15125
IgM	0.782	0.73225	0.7995	0.826	0.8005	0.79475	0.76825

B: Intradermal $1x10^7$ MVA 85A

	Day 0	Day 2	Day 7	Day 14	Day28	Day84	Day168
IgA	0.034	0.031	0.0465	0.04125	0.039	0.03875	0.04075
IgG	0.164	0.15325	0.14825	0.146	0.1445	0.2345	0.1475
IgM	0.86275	0.63375	0.76125	0.7635	0.748	0.76375	0.78125

OD values of serum IgA, IgG and IgM to Ag85A are shown. No significant differences were detected between the two vaccine groups or over the follow up period.
